# Epidemiology and Clinico-radiological features of Interstitial Lung Diseases

**DOI:** 10.12669/pjms.36.3.1046

**Published:** 2020

**Authors:** Saira Jafri, Naseem Ahmed, Nausheen Saifullah, Mehak Musheer

**Affiliations:** 1 Saira Jafri, MBBS. FCPS (Pulmonology) trainee, Jinnah Postgraduate Medical Centre, Karachi, Pakistan; 2 Naseem Ahmed, MBBS, FCPS. Assistant Professor, Jinnah Postgraduate Medical Centre, Karachi, Pakistan; 3 Nausheen Saifullah, MBBS, FCPS. Associate Professor, Jinnah Postgraduate Medical Centre, Karachi, Pakistan; 4 Mehak Musheer, MBBS. House Officer, Jinnah Postgraduate Medical Centre, Karachi, Pakistan; 5 Mubeen Iqbal, MBBS. House Officer, Jinnah Postgraduate Medical Centre, Karachi, Pakistan

**Keywords:** Interstitial lung disease (ILD), idiopathic pulmonary fibrosis (IPF), non-specific interstitial pneumonitis (NSIP), usual interstitial pneumonitis (UIP), gastro-esophageal reflux disease (GERD), Connective Tissue Disease (CTD)

## Abstract

**Objective:**

The literature on interstitial lung diseases is limited. The aim of this research was to make this entity of diseases more understandable to clinicians and general population of the region of Pakistan.

**Methods:**

We conducted a cross-sectional study on 253 Pakistani subjects who are a part of the hospital-based registry of JPMC. We performed statistical analyses on SPSS version 22.0. We included patients above 15 years of age who exhibited clinical clues and radiological signs of ILD during March 2016 through February 2018 and excluded those who were on tuberculosis treatment, suspected to be suffering from post-infection bronchiectasis, expectant females or had failed to follow-up.

**Results:**

There was a 2:3 male to female ratio. Mean age was 49.0±13.2 years. Majority were non-smokers. Idiopathic Pulmonary Fibrosis (IPF) was the commonest ILD (38.8%) followed by Non-Specific Interstitial Pneumonitis (NSIP) (15.1%). Most patients presented with dyspnea and dry cough and about half were clubbed (47.3%). Substantial IPF cases (52.6%) were suffering from GERD symptoms.

**Conclusion:**

IPF and NSIP were the major ILDs, GERD was the only predictor of IPF. This entity of lung diseases needs to be explored further to identify patterns of presentation and to make diagnosis at a manageable stage.

## INTRODUCTION

Interstitial lung disease (ILD) or diffuse parenchymal lung disease is characterized by inflammatory or fibrotic condition of the interstitium of lungs. There are more than 200 various manifestations of this condition based on clinical, radiological and histopathological findings.^1^ Epidemiologic studies and archive reports on diagnoses of ILD have often been dependent on International Classification of Diseases (ICD) 9th edition codes, chronological databases, or diagnosis by a single physician that exhibited variable accuracy.^2^-^4^ Consequently, the accurate diagnosis of ILD requires a wide multidisciplinary approach based on assessment by a pulmonologist, a radiologist and sometimes a histopathologist with comprehensive expertise in handling the cases of ILD.

From the studies, the most common ILDs are idiopathic pulmonary fibrosis (IPF), hypersensitivity pneumonitis (HP), sarcoidosis, ILD as a part of connective tissue disease (CTD), drug-induced ILD and pneumoconiosis.^5^-^7^ The majority of ILDs is idiopathic and includes the group of idiopathic interstitial pneumonias. In Pakistan, ILDs are among the leading causes of mortality i.e. 4.75%, while 14.56% die due to pneumonia influenza.^8^ The most common ILD, IPF, has a poor prognosis with median survival of 2-3 years from diagnosis^9^; for other forms of ILDs, prognosis depends on the underlying and/or accompanying disease(s) but may be similarly poor.

The spectrum of ILDs may be dissimilar from other regions of the world attributable to standards of living, atmospheric conditions, occupational exposures, lifestyle, smoking habits, socio-cultural norms and cultivation practices in addition to the genetic profile.^10^,^11^

Residence near industrial area, food centers, animal farms, highways and farming sites may be greater risk environments in progression of the disease.^12^-^14^ House architecture with poor ventilation, home appliances like air-conditioners, carpets and kitchen’s smoke may also influence the status of this group of diseases.^15^ A recent study fromIndia demonstrated need of research with biomarkers and genetic studies to evaluate contributory environmental factors in ILD registries.^16^

In our region, where pulmonary tuberculosis is the most prevalent of all the endemic respiratory ailments, the ILDs have been sparsely studied and are often underdiagnosed.

At JPMC, patients belonging to various South Asian ethnicities are treated. Hence, a research was conducted in the Pulmonology department, to ascertain the types and spectra of ILD’s on clinical and radiological bases.

## METHODS

It is a cross-sectional study on 253 Pakistani patients of either gender, with age >15 years, during a two years’ period i.e. from March 2016 to February 2018 in the Department of Pulmonology, Jinnah Postgraduate Medical Centre (JPMC), Karachi. This study was approved by the Institutional Review Board of JPMC (NO.F.2-81/2019-GENL/17644/JPMC, Dated February 7, 2019). The patients were confirmed as sufferers of ILDs on the bases of history, clinical examination and high resolution computed tomography (HRCT). Patients that were taking tuberculosis treatment, suffering from post-infection bronchiectasis, pregnant women and those who had failed to follow-up during the study period, were excluded. Recruitment in ILD clinic was done to elaborate ILDs, investigate for the specific type of ILDs by the respiratory specialist. Demographic data was evaluated. The information regarding comorbidities (diabetes, hypertension, ischemic heart disease, renal diseases, CNS ailments, rheumatological or hepatic disorders), lifestyle (smoking status, diet), habitat/locality (urban or rural, industrial or non-industrial), environmental risk factors (air-conditioner, carpet, ventilation, pets, chemical fumes and smoke), disease symptoms (cough, sputum, wheezing, breathlessness), other lung disorders (pneumonia, hay fever, tuberculosis, COPD, asthma) and drug use was thoroughly collected. GER was evaluated only on symptomatic basis i.e. reflux, indigestion, heartburn, sour taste and cough that disturbs sleep or a combination of these symptoms. Endoscopy was not done.

We performed statistical analyses on SPSS version 22.0 and studied frequencies of various ILD-types under categorized variables. The Chi-square testing was employed for possible correlation between the various categories. Logistic regression was performed for contributory factors of ILD. P-value ≤0.05 was considered statistically significant.

## RESULTS

**Fig.1:**
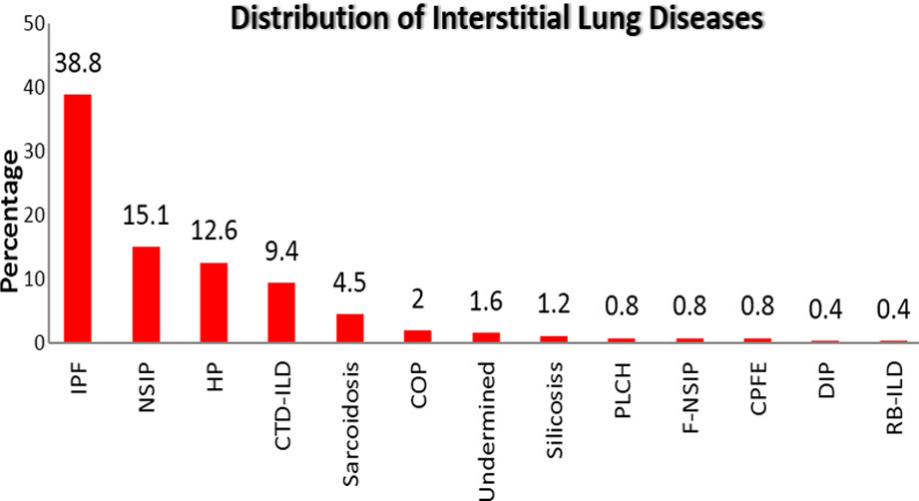
Distribution ILD subtypes.

There was clear female preponderance i.e. 169 (69%) versus 76 males (31%) exhibiting a 2:3 male to female ratio. Mean age was 49.0±13.2 years. IPF was the commonest ILD subtype that was found in 95 (38.8%) followed by NSIP (15.1%), CTD-ILD (9.4%), HP (12.6%), sarcoidosis (4.5%), Cryptogenic organizing pneumonia (2%), undetermined (1.6%) and silicosiss (1.2%) (Fig.1). Usual Interstitial Pneumonitis (UIP) was the most-common HRCT pattern exhibited by the disease (48.4%). Non-Specific Interstitial Pneumonitis (26.9%) and Chronic Hypersensitivity Pneumonitis (7%) were the next most prominent ones.

**Fig.2:**
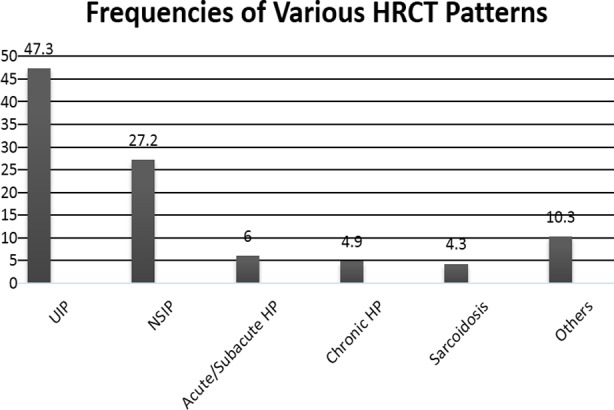
HRCT Patterns in ILD.

Dyspnea was the commonest of all symptoms (95.1%), followed by cough (86.1%) which was mostly dry. A significant number of subjects also presented with weight loss (52%), chest pain (42.9%), GERD (44.5%) and joint pain (38.4%).Commonest co-morbidity in the ILD subjects was found to be hypertension (28.2%), next to which were tuberculosis (19.6%) and diabetes mellitus (15.9%). 81.2% of the ILD subjects had never smoked. 12.7% were past smokers, whereas only 6.1% were current smokers.

With the help of this study, we observed that 36.3% of the subjects presented in the ILD clinic had been exposed to birds. Of these, 30% had confirmed exposure to parakeets/parrots, 28.9% to hen, 22.9% to pigeons, and 15.7% have been exposed to multiple birds.25.3% had a closed cooking area at their residencies, whereas 18% of the subjects had long-term contact with carpets. The prevalence of air conditioners, air coolers, and chimney exposures were 7.8%, 6.9% and 4.5% respectively. Hardly 23.2% of the ILD patients had a positive family history of some respiratory ailment most common of which was asthma (10.6%).

Among the signs, clubbing was found to be prevalent in 47.3% of the cases, of which, 38.7% was found in the IPF patients, 15.1% in patients with Idiopathic NSIP, 14.6% in the HP category, and merely 9% in the patients diagnosed with CTD. About 73% of patients had crepitations upon auscultation. Of these, 38.7% were the ones diagnosed with IPF, 15.1% with Idiopathic NSIP, 14.6% with HP, and 9% in those with CTD. It is also worth mentioning that 43.6% of the subjects were de-saturated during their first visit to the clinic.

Logistic regression analysis was performed to see the contributory effect of various factors on ILD subtypes. IPF and other ILDs was taken as binary variable and odd ratio with 95% CI of factors were calculated. GERD was the only predictor of IPF, while ulcers and biomass exposure were the marginally significant factors (p=0.06) shown in Table-III.

**Table-I:** Demographic features and symptoms of ILDs.

	IPF (n = 95)	NSIP (n = 37)	Sarcoidosis (n = 11)	CTD-ILD (n = 23)
*Gender*				
• Male	31 (32.6)	(29.7)	2 (18.2)	5 (21.7)
• Female	54 (67.4)	(70.1)	9 (81.8)	18 (78.3)
Age	52.0±12.3	47.1±13.0	49.4±10.1	46.1±17.1
*Symptoms*				
• Dyspanea	92 (96.8)	36 (97.3)	11 (100)	20 (87)
• Cough	85 (89.5)	32 (86.5)	9 (81.8)	19 (82.6)
• Phlegm	25 (26.3)	10 (27.0)	2 (18.2)	3 (13.0)
• Postnasal drip	19 (20.0)	12 (32.4)	1 (9.1)	4 (17.4)
• Weight loss	53 (55.8)	20 (54.1)	4 (36.4)	11 (47.8)
• Dysphagea	17 (17.9)	4 (10.8)	3 (27.3)	5 (21.7)
• Dryness	40 (42.1)	12 (32.4)	6 (54.5)	12 (52.2)
• Skin changes	13 (13.7)	2 (5.4)	1 (9.1)	3 (13.0)
• Pedal edema	22 (23.2)	10 (27.0)	1 (9.1)	3 (13.0)
• Hematuria	1 (1.1)	0 (0)	0 (0)	0 (0)
• Ulcers	26 (27.4)	12 (32.4)	4 (36.4)	14 (60.8)
• Hair-fall	15 (15.8)	3 (8.1)	1 (9.1)	4 (17.4)
• Raynauds	2 (2.1)	0 (0)	1 (9.1)	4 (17.4)
• Chest pain	39 (41.1)	14 (37.8)	5 (45.5)	11 (47.8)
• Joint pain	36 (37.9)	13 (35.1)	7 (63.6)	8 (34.8)
• GERD	50 (52.6)	17 (45.9)	4 (36.4)	10 (43.5)

**Table-II:** Associated illnesses of ILDs.

	IPF (n = 95)	NSIP (n = 37)	Sarcoidosis (n = 11)	CTD-ILD (n = 23)
Tuberculosis	17 (17.9)	10 (27.0)	3 (27.3)	4 (17.4)
Hypertension	31 (32.6)	14 (37.8)	2 (18.2)	6 (26.1)
Diabetes mellitus	18 (18.6)	8 (21.6)	0 (0)	3 (13.0)
Coronary Artery Disease	7 (7.4)	3 (8.1)	0 (0)	1 (4.3)
Congestive Cardiac Failure	1 (1.1)	0 (0)	0 (0)	0 (0)
Cerebro-Vascular Accident	1 (1.1)	1 (2.7)	0 (0)	1 (4.3)
Fits	1 (1.1)	0 (0)	0 (0)	1 (4.3)
Hepatitis	1 (1.1)	0 (0)	1 (2.7)	1 (4.3)
Kidney diseases	3 (3.2)	2 (5.4)	1 (9.1)	0 (0)
Pneumothorax	0 (0)	1 (2.7)	0 (0)	0 (0)
Pneumonia	8 (8.4)	2 (5.4)	0 (0)	3 (13.0)
Asthma	4 (4.2)	1 (2.7)	0 (0)	0 (0)
Pulmonary Hypertension	5 (5.3)	1 (2.7)	0 (0)	1 (4.3)
Obstructive Sleep Apnea	2 (2.2)	0 (0)	0 (0)	0 (0)
Rheumatological	4 (4.2)	3 (8.1)	2 (18.2)	5 (21.7)

**Table-III:** Contributory factor analysis of ILDs.

	IPF (n = 95)	Others (n=150)	Odd ratio (95% CI)	P-value
*Gender*				
• Male	31 (32.6)	45	1.47 (0.84-2.57)	0.179
• Female	54 (67.4)	115		
Dyspnea	92 (96.8)	141 (94.0)	1.96 (0.52-7.42)	0.315
Cough	85 (89.5)	126 (84.0)	1.62 (0.74-3.56)	0.227
Phlegm	25 (26.3)	34 (22.7)	1.22 (0.67-2.21)	0.515
Postnasal drip	19 (20.0)	36 (24.0)	0.79 (0.42-1.48)	0.465
Weight loss	53 (55.8)	76 (50.7)	1.23 (0.73-2.06)	0.434
Dysphagea	17 (17.9)	25 (16.7)	1.09 (0.55-2.15)	0.804
Dryness	40 (42.1)	55 (36.7)	1.26 (0.74-2.12)	0.395
Skin changes	13 (13.7)	19 (12.7)	2.63 (1.23-5.63)	0.818
Pedal edema	22 (23.2)	31 (20.7)	2.82 (1.51-5.26)	0.644
Ulcers	69 (72.6)	92 (61.3)	1.67 (0.96-2.92)	0.069
Hair-fall	15 (15.8)	27 (18.0)	0.85 (0.43-1.70)	0.655
Chest pain	39 (41.1)	66 (44.0)	0.89 (0.53-1.49)	0.650
Joint pain	36 (37.9)	58 (38.7)	0.97 (0.57-1.64)	0.904
GERD	50 (52.6)*	59 (39.3)	1.71 (1.02-2.88)	0.040
Tuberculosis	17 (17.9)	31 (20.7)	0.84 (0.43-1.61)	0.594
Hypertension	31 (32.6)	38 (25.3)	1.43 (0.81-2.51)	0.216
Diabetes mellitus	18 (18.9)	21 (14.0)	1.44 (0.72-2.86)	0.302
Visible Dust	34 (35.8)	55 (36.7)	0.96 (0.56-1.64)	0.889
Pillow	29 (30.5)	35 (23.5)	1.44 (0.81-2.57)	0.223
Biomass exposure	43 (45.3)	50 (33.6)	1.65 (0.99-2.80)	0.066
Closed cooking area	27 (28.4)	35 (23.3)	1.30 (0.73-2.34)	0.372
Change in house	20 (21.1)	26 (17.3)	1.27 (0.66-2.43)	0.468
Bird exposure	30 (31.6)	59 (39.9)	0.77 (0.45-1.33)	0.191
Caged/uncaged	16 (16.8)	28 (18.7)	0.88 (0.45-1.73)	0.717
Substance abuse	14 (14.7)	30 (20.0)	0.69 (0.35-1.38)	0.296
Current smoker	6 (6.3)	9 (6.0)	1.06 (0.36-3.07)	0.920
Ex-smoker	13 (13.7)	18 (12.0)	1.16 (0.54-2.50)	0.699

## DISCUSSION

The principal finding of the study reveals that IPF and NSIP are the major ILDs prevalent, 38.8% and 15.1% respectively while Singh S, et al.^17^ reported hypersensitive pneumonitis (HP) as the commonest ILD (47.3%) out of which about half (48.1%) had exposure to air coolers. Our findings were comparable to the likewise local studies^18^,^19^ where IPF was the commonest ILD and NSIP as a second frequent subtype of ILDs however, they found age above than 60 years as significant predictor of IPF while in our study GERD was the only significant factor in ILD patients. GERD was coupled with IPF as has been depicted by a number of studies already. One case-control research has shown that IPF patients are more susceptible to GER, hiatal hernia, and gastritis.^20^

Our registry exhibited additional findings related to environmental exposure in ILD patients and an ultimate effect of these features was recorded, which makes our study a step ahead to some local studies. Our study also dominates another paper by Sultana T, et al.^21^ with regards to studying magnitudes of ILDs, symptoms, associated illnesses and environmental exposure.

Usual interstitial pneumonitis was the most-common CT-scan pattern exhibited by the disease in this region followed by NSIP pattern which somewhere shows an unfortunate delay in diagnosis as well because many a times NSIP eventually progresses to UIP.^22^ Among the signs, clubbing was prevalent in 47.3% of the subjects, falling largely under fibrotic sort of ILD sub-category.^23^

Significant pulmonary hypertension (43%) was recorded in our patients, slightly exceeding the figures already published for its prevalence in connection with ILDs.^24^ In Pakistan, general physical health awareness and attitude towards screening, diagnosis and treatment of diseases is very casual. Lack of accessibility to quality healthcare services are the additional barriers in timely diagnosis and treatment. A recent study conducted in our set up recommends an intensive clinical suspicion of ILD earlier to avoid the misdiagnosis of TB in ILD cases because they reported that tuberculosis was treated before presentation in 38.35% of ILD patients, which is indeed a big number.^25^ In practice, the most common symptoms of cough and dyspnea tend to be unnoticed and misjudged due to smoking habits, aging or some infections, more commonly tuberculosis.

An important finding of our study is that the subjects had contact with parakeets/parrots, hens, pigeons and to multiple birds, compared to published data^26^ that barely shows noteworthy involvement of hens. Closed cooking area exposure was there in 25.3% and 18% of the subjects had had long-term contact with carpets. The commonness of air conditioners, air coolers, and chimney exposures are 7.8%, 6.9% and 4.5% respectively. Ansari M reported a dramatic finding of his study that out of 18 IPF & 5 HP diagnosed housewives were living in congested areas, 44.4% & 40% respectively had avian exposure due to home breeding or pets.^26^ In a prospective registry of ILD done in Saudi Arabia reported exposure sources for HP and were identified in 66.7% of the cases (pigeons and parrots = 7; sheep = 2; insecticides = 3; chemical paint = 1; and humidifiers = 1).^27^ Hypersensitivity Pneumonitis does not develop in each patient who is exposed to birds however it does remain the third most common cause of ILDs in our data. According to diagnostic radiological criteria hypersensitivity pneumonitis appears on HRCT mostly as homogenous symmetrical ground glass opacity in the middle and basal parts of lungs, along with centrilobular nodules and mosaic attenuation pattern. Also small volume mediastinal lymphadenopathy (generally 10-20 mm in short-axis diameter), with developing fibrosis, there can be reticulation, mainly in the middle portion of the lungs or fairly evenly throughout the lungs but with relative sparing of the extreme apices and bases. So whoever did not fulfil this criteria, despite birds’ exposure was not taken as HP. Nevertheless microscopic evidence of our diagnosis could not be provided as we did not perform biopsies which is a limitation of our work.

In our study, exposure of cigarette smoking was 18.8% that was relatively higher than a study (13.8%)^28^ done in India. Smoking although gradually damages the lungs and causes ILD, the environmental factors and other exposures should be identified and community awareness should be provided about these in order to cultivate a pollution-free and healthy environment.

There were 1.6% undetermined or unclassified ILDs consistent with the study in Saudi Arabia (1.8%) using the diagnostic criteria like ours and both these findings were relatively less than the reported prevalence of unclassifiable interstitial lung disease was 11.9% (95% confidence interval, 8.5–15.6%) in a systematic review.^29^

## CONCLUSION

With this registry, we could discover the most common ILD diagnoses, the affiliated signs, symptoms, comorbidities, and risk factors of the disease as well as the radiological features. This has led to a better understanding of the emerging entity in the region. For estimating prevalence in our population we would need data from other setups also that deal with ILDs and put all of them together. This subgroup of lung diseases needs to be explored even further to identify patterns of presentation and to make a diagnosis at a manageable stage. Public level awareness also needs to be spread so that measures are taken in order to prevent a significant number of ILDs and to avoid the debility.

### Authors’ Contribution:

**SJ:** Concept & design, interpretation of data, drafting, is responsible for integrity of research.

**NA:** Concept & design, critical revision of article.

**NS:** Final approval of the version to be published.

**MM, MI:** Acquisition and analyses of data. The manuscript has been read and approved by all the authors.
